# The proliferation, apoptosis, invasion of endothelial-like epithelial ovarian cancer cells induced by hypoxia

**DOI:** 10.1186/1756-9966-29-124

**Published:** 2010-09-10

**Authors:** Pengfei Zhu, Yanxia Ning, Liangqing Yao, Mo Chen, Congjian Xu

**Affiliations:** 1 Department of Gynecology, Obstetrics & Gynecology Hospital, Fudan University, 419 Fangxie Rd, Shanghai, 200011, China; 2 Department of Obstetric & Gynecology, Shangyu City Hospital, 517 Shimin Blvd Baiguan St, Shangyu, Zhejiang Province, 312000, China; 3 Department of Physiology & Pathophysiology, Shanghai Medical College, Fudan University, 138 Yixueyuan Road, Shanghai, 200032, China

## Abstract

**Background:**

Epithelial ovarian cancer is one of the most malignant cancers in women because metastasis occurs in the most of patients by the time of diagnosis. Cancer cells have strong capacity to form angiogenesis or vasculogenic mimicry, which plays the major role in its malignant phenotype. Vasculogenic mimicry might contribute to the failure of the angiogenesis-targeted therapy strategies. Under the microenvironment of the tumor, hypoxia is the most common phenomena because of the vast energy and oxygen consuming. In the present study, the endothelial-like cells induced by hypoxia from SKOV-3 and ES-2 ovarian cancer cells were harvested to investigate the changes in their biological behaviors.

**Methods:**

The endothelial-like cells from SKOV-3 and ES-2 cells were harvested by laser capture microdissection. The biological behaviors of the endothelial-like cells, including proliferation, cell cycle, apoptosis, invasion and telomerase activity were determined by MTT, FCM, Transwell chamber and TRAP-ELISA methods. HIF-1α is the most important factor for the behavior changes under hypoxic condition. Some other genes relative to biological behaviors are also changes following the changes of HIF-1α. In order to elucidate the underlying mechanisms for these changes by hypoxia, the relative genes expressions including HIF-1α, CyclinD1, Flk-1, VEGF, p53 and V-src were determined by real-time PCR.

**Results:**

SKOV-3 and ES-2 cells were resistant to hypoxia by adoption of proliferation, apoptosis, differentiation and invasion. Combined with other studies, the more poorly cancer cells differentiate, the more strongly cells are resistant to hypoxia, the more possible to form vasculogenic mimicry. The changes in the expression of HIF-1α, and HIF-1α-dependent VEGF, Flk-1, Cyclin D1, and HIF-1α-independent p53 have been involved in this process.

**Conclusions:**

HIF-1α took an important role in the behavioral changes of SKOV-3 and ES-2 cells by hypoxia. At the same time, other mechanisms were also involved in this process.

## Background

Epithelial ovarian cancer (EOC) has the ~50% mortality rate, making it the leading cause of death from gynecological cancers [1,2]. In most patients, metastasis occurs within the peritoneum by the time of diagnosis. Although the cellular and molecular mechanisms of tumor growth and metastasis are not completely understood, it is established that formation and growth of new blood vessels is critical for tumor survival, growth, and expansion [3]. Numerous studies have demonstrated that the more vasculogenesis, the more malignant of the tumors. Thus, efforts to reduce the growth and spread of ovarian cancer have recently focused on angiogenesis because they are dependent in part on the formation of adequate vascular support [4], which means forming or sprouting of new endothelium-lined vessels from preexisting vessels [5].

The traditionally recognized mechanism for tumor vasculature and perfusion has been thought to be endothelial cells-lined vascular networks [6]. However, recent study has found that some aggressive tumor cells generate vasculogenic-like channels in the absence of endothelial cells or fibroblasts [7]. The formation of the patterned microcirculation is termed vasculogenic mimicry (VM), which indicates the process by which aggressive tumor cells are able to generate not-endothelial cell-lined channels delimited by extracellular matrix *in vitro *[7-9]. That's the reason why it is difficult to control ovarian cancer with angiogenesis-targeted therapy strategies [9] which have no positive effect on such vasculogenesis.

Hypoxia is one of the major important factors in angiogenesis descried by Folkman for it is associated with resistance to chemo- and radio-therapies. The development of tissue hypoxia is characteristically observed as malignant tumor rapidly increase in size. Such hypoxic conditions exert selective pressure on cancer cells, and the ability of tumor cells to survive in a hypoxic microenvironment has been associated with a poor prognosis and resistance to therapy [10]. One of the most critical and best characterized responses to hypoxia is the induction of vascular endothelial growth factor (VEGF), and hypoxia-inducible factor-1 (HIF-1) is a well-established mediator in this process. Our previous studies have demonstrated that the ovarian cancer cells could be induced into endothelial-like cells which have the specific characteristics of endothelial cells at the condition of hypoxia *in vivo *and *in vitro *[11-13], in which HIF-1α played a vital role.

As it is known that the endothelial-like cells (EL) origin from cancer cells are different from the endothelial cells. However, the detailed difference and the mechanisms are not well understood. In the present study, we set out to determine some biological behaviors of the ELs from two malignant ovarian cancer cell lines, SKOV-3 and ES-2, such as the proliferation, cell cycle, apoptosis, the activity of telomerase and invasion. At the same time, we compared these biological behaviors with traditional endothelial cell, human umbilical vein endothelial cell (HUVEC) and the original cancer cells. Further, we tried to explore the underlying mechanisms by detection the expression of some relative genes.

## Methods

### Cell culture

Human epithelial ovarian carcinoma cell lines SKOV-3 and ES-2 were purchased from American Type Culture Collection (ATCC, Manassas, VA), and were maintained in McCoy's 5a. Primary human umbilical vein endothelial cells (HUVEC) were isolated from umbilical vein and cultured as described previously [14]

### Three-dimensional cultures and hypoxic treatment

Thirty microliters of Matrigel (B&D, Bedford, MA) were dropped onto each glass coverslip in a 12-well culture plate and polymerized for 1 h at room temperature, followed by 30 min's incubation at 37°C in a humidified 5% CO_2 _incubator, as described previously [15]. Tumor cells (1 × 10^4^) were seeded onto the three-dimensional gel. The medium supplied with 15% FBS was changed every 36 h. Hypoxic condition was created by flushing 5% CO_2 _and 95% N_2 _through a modified chamber (Mitsubishi, Japan), until O_2 _concentration was reduced to 1%, measured with a Mini oxygen meter. The culture system was sealed and incubated at 37°C [16]. The cells were treated with 50 nM Sirolimus (Sigma, St. Louis, MO) in DMSO to inhibit the role of HIF-1α under hypoxia when necessary.

### Proliferation assay

For the proliferation assay, 1 × 10^4 ^SKOV-3, ES-2 and HUVEC cells, were seeded into a flat bottom 96-well plate and incubated at 37°C for 3 and 7 d under normoxia or hypoxia (1% O_2_) respectively, prior to the addition of 20 μL of MTT solution (5 mg/ml in PBS). After incubated for additional 4 h at 37°C, absorbance at 490 nm was measured with a multi-function reader (Tecan GENios, Zurich, Switzerland) to determine cell viability.

### Cell cycle and apoptosis assay

Cell cycle and apoptosis assay were performed on cells with or without hypoxia treatment (for 3 or 7 d) to determine whether hypoxia regulates the growth phase and apoptosis of epithelial ovarian cells. Cells were trypsinized and centrifuged at 300 × g (1000 rpm) for 5 min, then resuspended (1 × 10^6 ^cells/ml) and fixed with 70% ice-cold ethanol for 30 min, followed by centrifuged, washed and resuspended in 500 μl PBS contained 10 μl of DNase free RNase (final concentration is 1‰). After 30 min incubation, pyridine iodide (PI, 0.05 mg/ml) was added to the solution to incubate for an additional 15 min in the dark and filtered by a nylon mesh to remove cell clusters. The fluorescence of PI was measured using FACS Calibur Flow Cytometer (Becton-Dickinson, San Jose, CA). Cell subpopulations in G0/G1, S and G2/M phases and apoptosis were calculated by gating analysis based on differences in DNA content. At least 20000 cells were analyzed per sample. Cell proliferation characters were indexed by the ratio in S-phase.

### Invasion assay

Invasion assays were performed in a 24-well transwell chamber (Costar, Bodenheim, Germany) as previously described [17]. Briefly, the 8 μm pore inserts were coated with 15 μg of Matrigel. Cells were seeded to coated filters (5 × 10^4 ^cells/filter) in 200 μL of serum-free medium in triplicate. Another 500 μL of serum-free media was added in the lower parts of the chambers. After 7d's incubation under hypoxia, the upper Matrigel coated surface was wiped off using a cotton swab. Cells migrated through the filters were fixed, stained with Giemsa (Sigma, St. Louis, MO), photographed, and counted.

### Laser capture microdissection

Fifteen microliters of Matrigel were mounted on ethylene vinyl acetate (EVA) membrane (Leica, Wetzlar, Germany) with frame instead of coverslip in 9-cm dishes and treated to establish three-dimensional culture as described above. The density of tumor cells seeded onto gel was adjusted to 1 × 10^5^. After 7 d, samples on EVA membrane were washed with PBS-DEPC and air-dried, channels formed by endothelial-like cells (ELs) were selected by microscopy and microdissected with laser capture microdissection (LCM) system (Leica). About 1,500-2,000 ELs were laser-captured from each EVA membrane. The cells were immersed in digestion buffer for quantitative real-time reverse transcription polymerase chain reaction (RT-PCR) and telomerase activity assay.

### Quantitative real-time RT-PCR

Total RNA was extracted from 2 × 10^4 ^cells (including HUVEC, SKOV-3, SKOV-3 EL, ES-2, ES-2 EL, or the SKOV-3 or ES-2 cells treated by 50 nM Sirolimus) using TRIzol reagent (Invitrogen, Carlsbad, CA). Aliquots of RNA were reverse transcribed to cDNA using a Superscribe First-Strand synthesis system (Invitrogen). Real-time PCR analysis was performed to quantify mRNA expression of HIF-1α, VEGF, Flk-1, Cyclin D1, p53, and V-src by a Rotor-Gene3000 PCR system (Corbett, Australia) using SYBR-Green PCR Master mix (Qiagen, Hilden, Germany). The PCR reaction consisted of 12.5 μl of SYBR-Green PCR Master mix, 1.0 μl of forward and reverse primers (0.4 μM final concentration), and 2.0 μl of 1:10-diluted template cDNA in a total volume of 25 μl. Amplification was initiated at 50°C for 2 min, 95°C for 70 sec, followed by 40 cycles of 95°C for 20 sec, 58°C for 20 sec, and 72°C for 30 sec. To verify only a single product produced, a dissociation protocol was added after thermocycling. The assay included a no-template control, a standard curve of four serial dilution points (in steps by 10-fold) of a cDNA mixture. All data were controlled by Rotor-Gene software (version 6.0) for quantity of RNA input, an endogenous reference gene (β-actin) was performed as control in the same reverse transcription reaction. Data were presented as the means ± S.E from three separate experiments. The primers used in this experiment were shown in Table 1. All primers were designed using Primer3 web software (Whitehead Institute, Cambridge, MA) and were synthesized by Sangon Biological Engineering Technology and Service Co., Ltd. (Shanghai, P.R. China).

**Table 1:** The sequences of the primers used in the experiment

Gene	Sense	Antisense	Product (bps)
HIF1α	TGCACAGGCCACATTCACGT	GTTCACAAATCAGCACCAAGC	97
Flk-1	ACAGTGGTATGGTTCTTGCCTCA	GTAGCCGCTTGTCTGGTTTGA	140
VEGF	TCACCAAGGCCAGCACATAG	GGGAACGCTCCAGGACTTAT	166
Cyclin D1	GATGCCAACCTCCTCAACGAC	CTCCTCGCACTTCTGTTCCTC	171
V-src	CACTCGCTCAGCACAGGACAG	AGAGGCAGTAGGCACCTTTCG	196
P53	GCTGCTCAGATAGCGATGGTC	CTCCCAGGACAGGCACAAACA	298
β-actin	CCTGTACGCCAACACAGTGC	ATACTCCTGCTTGCTGATCC	211

### Telomerase activity assay

The telomerase activity of all the cells (including HUVEC, SKOV-3, SKOV-3 EL, ES-2, ES-2 EL, or the SKOV-3 or ES-2 cells treated by 50 nM Sirolimus) was tested by telomerase repeat sequence amplification-enzyme linked immunosorbent assay (TRAP-ELISA) using the kit from Huamei Biotechnology Co., Ltd. (Shanghai, China) according to the manufacturer's instruction.

### Statistical analysis

*ANOVA *analysis or paired-samples *t-test *were performed to identify differences, using SPSS11.5 statistical software (Lead, US). Statistical significance was assumed at P < 0.05, P-values are presented as two-tailed.

## Results

### The morphology of the endothelial-like cells from ovarian cancer shows similarities to HUVEC endothelial cells

**Figure 1:**
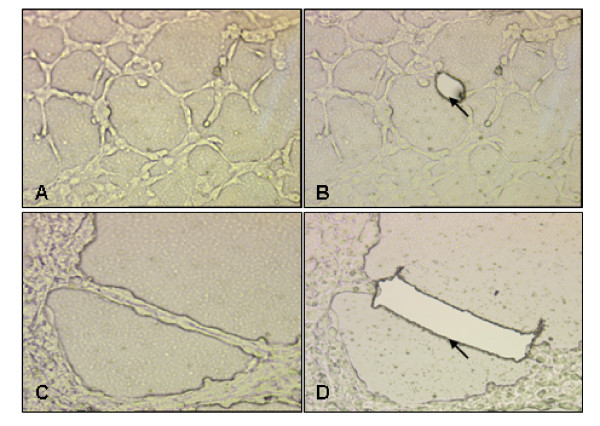
**The morphology of the ELs from ovarian cancer induced by hypoxia and microdissected by LCM**. The ovarian cancer cells were cultured in 3-dimisonal Matrigel system on EVA membrane under hypoxia for 7 d before harvest. The pictures were taken under the light microscope. **A **and **B**. The original and after microdissected by LCM of the single cell. **C **and **D**. The original and after microdissected by LCM of the grouped cells. Magnification X200. Arrow: The morphology of the cells after microdissection.

To investigate the morphology of the endothelial-like cells from ovarian cancer induced by hypoxia, the SKOV-3 and ES-2 cells were cultured in the 3-dimensional Matrigel system on EVA membrane under 1% O_2 _for 7 d before harvested by LCM. The morphology of the endothelial-like cells induced by hypoxia were pictured by microscope and shown in Figure 1. As it shown, after incubated under hypoxia, the ovarian cancer cells extended and reshaped, developed ELs and connected with each other (A and B), eventually forming network structures and channels (C and D). The original and microdissected by LCM of the single cell were shown in Fig. 1A and 1B, Fig. 1C and 1D indicated the original and microdissected grouped cells.

### The biological behaviors such as proliferation, cell cycle, apoptosis and invasion of SKOV-3, ES-2 and HUVEC cells are changed by hypoxia

**Figure 2:**
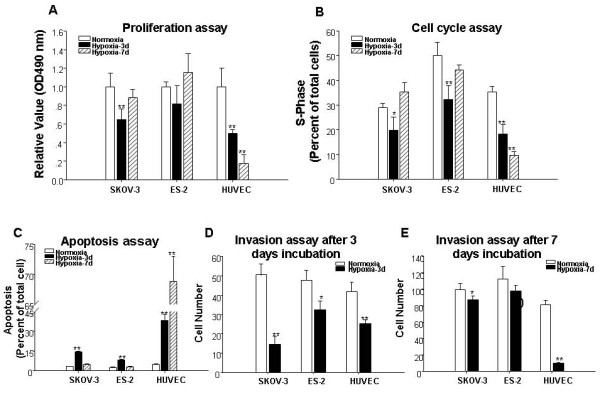
**The proliferation, cell cycle, apoptosis, invasion of SKOV-3, ES-2 and HUVEC cells induced by hypoxia**. The SKOV-3, ES-2 and HUVEC cells were cultured for 3 or 7 d in normoxia or hypoxia conditions before proliferation, cell cycle (S-phage), apopotosis and invasion detected by MTT, FCM (for cell cycle and apoptosis) and Transwell as shown in methods. **A**. The proliferation of three cells by MTT. **B**. The S-phase ratio in three cells by FCM. **C**. The apoptosis of three cells detected by FCM. **D **and **E**. The numbers of cells invasion through the membrane indicated by Transwell after incubated for 3 days (D) or 7 days (E). Data were shown in Mean ± S.D. from three separate experiments with the similar result. * and ** indicates P < 0.05 and P < 0.01 vs. Normoxia.

In order to elucidate the biological behaviors changes in SKOV-3, ES-2 and HUVEC cells by hypoxia, the proliferation, cell cycle, apoptosis and invasion were detected by MTT, FCM and transwell chamber after induced by hypoxia for 3 or 7 d. As shown in Fig. 2A, the proliferation of SKOV-3 was inhibited significantly on 3rd d while there was no difference after 7d's incubation. As for the proliferation of ES-2 cells, there has no significant difference after incubation under hypoxia. The proliferation of HUVEC cells were inhibited by incubation under hypoxia for 3 d and further inhibited after 7 d's incubation.

The percent of cells in S-phase and apoptosis after incubation for 3 or 7 d under hypoxia were shown in Fig. 2B and 2C. As they shown, in the case of SKOV-3 and ES-2 cells, the percent in S-phase were decreased and those of apoptosis were increased after 3 d's incubation, however, there had no difference in S-phase and apoptosis after 7 d's incubation of the two cell lines. On the other hand, the percent of S-phase of HUVEC cells was decreased and that of apoptosis was increased after both 3 and 7 d's incubation.

**Figure 3:**
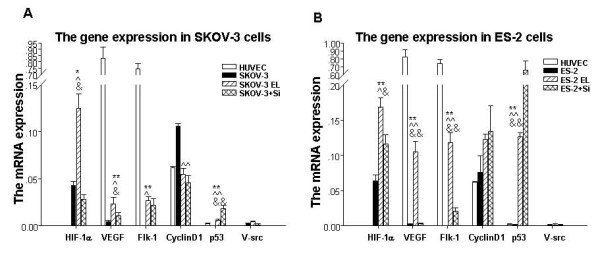
**The genes expression in SKOV-3, ES-2, ELs from cancer cells and HUVEC induced by hypoxia**. The SKOV-3, ES-2 and HUVEC cells were cultured for 7 d in normoxia or hypoxia conditions before harvested for the expression of HIF-1a, VEGF, Flk-1, CyclinD1, p53 and V-src genes detected by Real-time PCR. **A**. The genes expression in SKOV-3 and relative cells by Real-time PCR. **B**. The genes expression in ES-2 and relative cells by Real-time PCR. **SKOV-3 EL: **the endothelial-like cells induced from SKOV-3 cells; **SKOV-3+Si: **the SKOV-3 cells treated by Sirolimus under hypoxia; **ES-2 EL: **the endothelial-like cells induced from ES-2 cells; **ES-2+Si: **the ES-2 cells treated by Sirolimus under hypoxia; *, ^, and & indicates that P < 0.05 vs.HUVEC, SKOV-3 (or ES-2) and SKOV-3+Si (or ES-2+Si); **, ^^, and && indicates that P < 0.01 vs.HUVEC, SKOV-3 (or ES-2) and SKOV-3+Si (or ES-2+Si).

The numbers of cell migrated through basement membrane of the transwell chamber were shown in Fig. 3D (after 3 d's incubation) and 3E (after 7 d's incubation). Compared to normoxia control, the numbers decreased significantly in SKOV-3 after 3 and 7 d's incubation under hypoxia while it decreased significantly in ES-2 only after 3 d's incubation. The numbers of HUVEC cells were decreased significantly after both 3 and 7 d's incubation.

### The activities of telomerase of SKOV-3, ES-2 and HUVEC cells are changed by hypoxia

In order to study the malignant of the ovarian cancer cells, the activities of telomerase of SKOV-3, ES-2 and HUVEC cells incubated under hypoxia, normoxia or hypoxia with Sirolimus were detected by TRAP-ELISA. As shown in Table 2, the activities of telomerase were positive in all the SKOV-3 endothelial-like cells, SKOV-3 under normoxia or with Sirolimus. The activities of telomerase were negative in ES-2 endothelial-like cells and ES-2 with Sirolimus but positive in ES-2 under normoxia. As we expected, the activity of telomerase was negative in HUVEC cells.

**Table 2:** The activity of telomerase in different cells

CELLS	RESULT
HUVEC	-
SKOV-3	+
SKOV-3 EL	+
SKOV-3+Si	+
ES-2	+
ES-2 EL	-
ES-2+Si	-

**SKOV-3 EL: **the endothelial-like cells induced from SKOV-3 cells; **SKOV-3+Si: **the SKOV-3 cells treated by Sirolimus under hypoxia; **ES-2 EL: **the endothelial-like cells induced from ES-2 cells; **ES-2+Si: **the ES-2 cells treated by Sirolimus under hypoxia.

### The different expression of HIF-1α, CyclinD1, VEGF, Flk-1, p53 and V-src mRNA in SKOV-3, ES-2 and HUVEC cells after incubation under hypoxia

In order to elucidate the underlying mechanisms for the biological behaviors changes of the ELs by hypoxia, the mRNA expression of HIF-1α, CyclinD1, VEGF, Flk-1, p53 and V-src in SKOV-3, ES-2 and HUVEC cells incubated under hypoxia, normoxia or hypoxia with Sirolimus were detected by Real-time PCR. The genes expression mentioned above in SKOV-3 and SKOV-3 relative cells were shown in Fig. 3A and Fig. 3B indicated the genes expression in ES-2 and ES-2 relative cells.

As shown in Fig. 3, HIF-1α mRNA expression in both of the two tumors' ELs was significantly higher than that in the cells under normoxia and with Sirolimus, and than that in HUVEC cells.

VEGF mRNA expression in both of the two tumors' ELs was significantly higher than that in the cells under normoxia and with Sirolimus, but was greatly lower than that in HUVEC cells.

Flk-1 mRNA expression in both of the two tumors' ELs was significantly higher than that in the cells under normoxia, but was greatly lower than that in HUVEC cells. On the other hand, Flk-1mRNA expression in ES-2 endothelial-like cells was significantly higher than that in cells treated with Sirolimus, however, there was no difference in Flk-1 mRNA expression between SKOV-3 endothelial-like cells and SKOV-3 cells treated with Sirolimus.

Cyclin D1 mRNA expression in both of the two tumors' ELs was greatly lower than that in the cells under normoxia, while there was no difference in Cyclin D1 mRNA expression in the cells treated with Sirolimus and HUVEC cells.

p53 mRNA expression in both of the two tumors' ELs was significantly higher than that in the cells under normoxia and in HUVEC cells, however, there was no significant changes after treated with Sirolimus.

V-src mRNA didn't express in all kinds of cells under hypoxia or normoxia.

## Discussion

In the present study, we induced two ovarian cancer cell lines, SKOV-3 and ES-2, to endothelial-like cells by hypoxia and harvested the ELs by LCM. On the base of our previous study [11], the ELs have the specific characteristics of endothelial cells, such as expressing CD34, vWF and uptaking acLDL. Here, we detected the biological behaviors of the ELs and compared with the HUVEC endothelial cells and the original cancer cells.

As shown in the results, under the condition of hypoxia, the cancer cells' growth was inhibited in the short period (3 d), however, after the long-time hypoxia (7 d) incubation, the cells were recovered to grow. The results of the proliferation assay, cell cycle and apoptosis assay demonstrated these. HUVEC, on the other hand, could not endure hypoxia, which showed inhibited proliferation, reduced S-phase ratio, and increases in apoptosis under the condition of hypoxia. As indicated by previous studies [10,18], the more aggressive of the cancer, the more strongly the cells could resistant to hypoxia. Under the condition of hypoxia, the cancer cells could change some characteristics into ELs to form VM, and then the tumor could perfuse itself independent of angiogenesis. Tumors exhibiting in VM related to more aggressive tumor biology and increased tumor-related mortality [19,20]. Invasion through the basement membrane is one of the features of the aggressive tumor. Under the condition of hypoxia, the SKOV-3 and ES-2 ovarian cancer cells reduced the ability to invasion at first and then recovered to normal level after long-time hypoxia.

Telomerase, an enzyme complex that binds the chromosome ends (telomeres) and maintains telomere length and integrity, is present in germ cells, proliferative granulose cells, germline stem cells, and neoplastic cells in the ovary, but is absent from differentiated or aged cells. Activation of telomerase in the ovary underpins both benign and malignant cell proliferation. Normally, high levels of telomerase activity are a hallmark of cancer, including ovarian epithelial carcinoma [21]. Accumulating data indicate that telomerase activation is an early event in ovarian carcinogenesis [22-25]. As expected, the telomerase activities were positive in both SKOV-3 and ES-2 cells and negative in HUVECs. At the same time, the telomerase activities in ELs from SKOV-3 cells with or without Sirolimus treatment were also positive while those in ELs from ES-2 cells with or without Sirolimus were negative. The difference of telomerase activity between the two ELs may contribute to the different proliferative behaviors of the two cells.

To explore the underlying mechanisms of the SKOV-3 and ES-2 changed to ELs by hypoxia treatment, we detected the expression of some relative genes in the SKOV-3, ES-2, SKOV-3 ELs, ES-2 ELs, with or without Sirolimus, and HUVECs. As Fig. 3 shown, compared with the original cancer cells, the ELs represented the elevated HIF-1α, VEGF, VEGF receptor-2 (Flk-1) and p53 mRNA expression, while the expression of Cyclin D1 was decreased. Our and others' studies have indicated that HIF-1α played a vital role for the angiogenesis and VM under hypoxia [11,26-28]. To determine the origin of the change in VEGF and Flk-1 expression, we used the Sirolimus to inhibit the activity of HIF-1α. Sirolmus, known as rapamycin, is proved to be as the inhibitor of HIF-1α [26,29,30]. Consistent with other researches, the changes in the expression of VEGF, Flk-1 and Cyclin D1 were HIF-1α transcriptional dependent [10,31]. However, the change in the expression of p53 was HIF-1α transcriptional independent.

## Conclusion

In summary, the ovarian cancer cells could be induced into ELs which seemed similarly to progenitor endothelial cells by hypoxia. After induced, the ELs would get some characteristics of endothelial cells and would lose some malignant characteristics of the original cancer cells. The increased expression of HIF-1a, and HIF-1α depended VEGF and Flk-1 might contribute to the VM and the vasculogenesis. During the transition, HIF-1α took an important role in the molecular mechanisms, while there still has other HIF-1α-independent mechanism in this process.

## List of abbreviations

EL(s): endothelial-like cell(s); EOC: epithelial ovarian cancer; EVA: ethylene vinyl acetate; HIF-1: hypoxia-inducible factor-1; HUVEC: human umbilical vein endothelial cell; LCM: laser capture microdissection; RT-PCR: reverse transcription polymerase chain reaction; TRAP-ELISA: telomerase repeat sequence amplification-enzyme linked immunosorbent assay; VEGF: vascular endothelial growth factor.

## Competing interests

The authors declare that they have no competing interests.

## Authors' contributions

PZ carried out the proliferation, cell cycle and apoptosis assay, participated in drafted the manuscript. YN carried out the invasion experiment, participated in experiment design and drafted the manuscript. LY conceived of the study, participated in its design and coordination, performed the statistical analysis and helped to draft the manuscript. MC carried out the telomerase activity assay, participated in the draft preparation. CX participated in the design of the study and performed the statistical analysis. All authors read and approved the final manuscript.

## Authors' informations

PZ, M.D., medical master candidate, Dept. Gynecology, Obstetrics & Gynecology Hospital, Fudan University; senior medical registrar, Dept. Obstetric & Gynecology, Shangyu City Hospital; YN, M.D. & Ph.D., assistant professor, Dept. Physiology & Pathophysiology, Shanghai Medical College, Fudan University; LY, M.D. & Ph.D., associate professor & medical consultant, Dept. Gynecology, Obstetrics & Gynecology Hospital, Fudan University; MC, M.B., medical master candidate, Dept. Gynecology, Obstetrics & Gynecology Hospital, Fudan University; CX, M.D. & Ph.D., professor & senior medical consultant, Dept. Gynecology, Obstetrics & Gynecology Hospital, Fudan University.
